# Evaluating milk quality indicators in the era of antimicrobial stewardship: A study of tennessee dairy farms (2011–2023)

**DOI:** 10.1371/journal.pone.0338386

**Published:** 2025-12-31

**Authors:** Roderick T. Salvador, Jessica Vidlund, Chika C. Okafor

**Affiliations:** 1 College of Veterinary Science and Medicine, Central Luzon State University, Nueva Ecija, Philippines; 2 College of Veterinary Medicine, University of Tennessee-Knoxville, Knoxville, Tennessee, United States of America; University of Illinois Urbana-Champaign College of Veterinary Medicine, UNITED STATES OF AMERICA

## Abstract

This study evaluated temporal and seasonal trends in milk quality parameters: somatic cell count (SCC), fat, and protein content among Tennessee dairy farms from 2011 to 2023, with reference to the 2017 implementation of the Veterinary Feed Directive (VFD). A total of 8,357 herd-month records from the Tennessee Dairy Herd Improvement Association (DHIA) were analyzed using linear mixed-effects models to assess year, season, and policy-period effects while accounting for clustering by herd size. Results showed a significant decline in SCC (−17.5%, *p* < 0.001) and significant increases in milk fat (+8.48%) and protein (+2.95%) over the study period. Seasonal variation was pronounced, with higher SCC and lower components during summer months. Although improvements in milk quality coincided with the post-VFD period, the directive primarily targeted antimicrobials in feed and water rather than intramammary therapy; thus, observed changes likely reflect continued advances in herd management, nutrition, and genetics rather than a direct regulatory effect. These findings highlight the role of environmental and management adaptation in sustaining milk quality under evolving production and policy contexts.

## Introduction

Milk quality is a key determinant of dairy profitability, consumer confidence, and product competitiveness [1–3]. High-quality milk enhances cheese yield, flavor stability, and shelf life, while poor quality reduces processing efficiency and can incur economic penalties [1]. For dairy producers in Tennessee, sustaining high milk quality is essential to remain competitive within regional and national markets.

Somatic cell count (SCC), milk fat, and protein content are the principal indicators of milk quality. SCC, measured as the number of leukocytes in bulk-tank milk, reflects udder health; elevated counts indicate inflammation typically associated with subclinical or clinical mastitis [4]. The U.S. regulatory threshold for Grade A milk is 750,000 cells/mL [5], though most processors apply stricter limits below 400,000 cells/mL [2]. Fat and protein determine both the nutritional and economic value of milk fat influences flavor and butter yield, while protein, particularly casein, drives cheese-production efficiency [6,7].

In 2017, the U.S. Food and Drug Administration introduced the Veterinary Feed Directive (VFD GFI #213), which withdrew growth-promotion claims and required veterinary oversight for all medically important antimicrobials administered in feed or water [8]. The guidance excluded intramammary tubes, injectables, and other non-feed dosage forms. Those products remained available over-the-counter until FDA GFI #263 (2021) required prescription-only marketing, a transition completed after the close of our study window [8,9]. Because lactating cows are rarely medicated via medicated feed or water and such use would risk antimicrobial residues in milk, GFI #213 did not reduce antimicrobial exposure at the udder level. National data confirm that sales and distribution of intramammary antimicrobials have remained stable over the past decade [9]. Any temporal association between the 2017 policy and milk-quality improvements must therefore be viewed as an indirect, management-mediated effect (e.g., more frequent veterinary visits, improved hygiene protocols) rather than as a direct pharmacological consequence of reduced antimicrobial use.

Any relationship between the VFD’s implementation and milk-quality indicators should therefore be interpreted as indirect and temporal rather than causal. The policy may have encouraged enhanced veterinary oversight, improved record-keeping, and preventive herd management, indirectly supporting better hygiene and animal-health vigilance. Observed changes in milk-quality parameters such as SCC, fat, and protein are more plausibly attributed to advances in management, genetics, and environmental adaptation than to a pharmacological effect of the VFD itself.

Tennessee provides a relevant context for examining these relationships. The Tennessee Quality Milk Initiative (TQMI) and the Southeast Quality Milk Initiative (SQMI), both launched before the VFD reduced herd-level SCC by approximately 25% between 2012 and 2017 [10], demonstrating the effectiveness of management-based programs in improving milk hygiene [11]. However, the extent to which these improvements persisted beyond 2017 remains unclear.

The relationships among policy, management, environment, and milk-quality outcomes are illustrated in S1 File. The VFD represents a policy-level intervention that may indirectly influence farm management behaviors such as milking hygiene, veterinary engagement, record-keeping, and biosecurity. These practices can, in turn, affect the immediate environment of dairy herds through factors such as stall cleanliness, ventilation, and heat-stress control. Broader environmental conditions, particularly seasonal temperature and humidity, exert direct effects on udder health and milk composition. In the conceptual framework, solid arrows denote relationships supported by literature or observed seasonality, whereas dashed arrows indicate hypothesized pathways that were not empirically evaluated but provide context for the descriptive trends analyzed in this study.

This study therefore aimed (1) to describe trends in SCC, fat, and protein content among Tennessee dairy farms from 2011 to 2023; (2) to examine seasonal variations in these parameters as indicators of environmental influence; and (3) to evaluate temporal changes surrounding the 2017 implementation of the VFD, recognizing that any associations observed are descriptive rather than causal.

## Materials and methods

### Study population and data source

This study was a retrospective longitudinal analysis using secondary data from Dairy Herd Improvement Association (DHIA). The dataset spanned January 2011 to December 2023 and included all available herd-level milk-quality records from Tennessee dairy farms enrolled in the DHIA testing program during this period. Participation in DHIA testing is voluntary and may underrepresent very large herds that rely on automated on-farm metering systems rather than centralized testing.

Milk composition and udder-health data were originally collected at the individual cow level during routine test-day sampling and subsequently aggregated by DHIA into monthly herd-level summaries prior to data release. Aggregation involved calculating the mean SCC, milk-fat percentage, and milk-protein percentage across all test-day samples for each herd within a given month. These herd-month averages constituted the analytical unit for all statistical analyses.

The final dataset comprised 8,357 de-identified herd-month observations, representing repeated measurements from Tennessee dairy herds over a 13-year period. Across these records, the monthly pool of lactating cows contributing data typically ranged from approximately 10,000–45,000 head. The DHIA requires at least 10,000 cows per state-month for reporting validity, but exact monthly cow counts were not released to the research team. Because farm identifiers were anonymized and not stable across years, the number of unique farms contributing to each month or year could not be enumerated. According to DHIA classification, the median herd size was 249 lactating cows (mean = 320), reflecting the predominance of medium-sized operations among DHIA participants.

As part of internal data quality assurance, DHIA excluded any state–month combination with fewer than four contributing herds or fewer than 10,000 lactating cows, as well as incomplete or biologically implausible records (e.g., missing SCC, fat, or protein values). These procedures were performed prior to anonymization and data release. No additional filtering was applied by the research team.

All data were de-identified before transfer to ensure confidentiality. Farm identifiers were replaced with numeric codes, and individual cow-level data were not accessible. Because DHIA participation is voluntary, the analytic cohort may not capture all Tennessee dairies. The state’s dairy sector has undergone consolidation, with fewer, larger, and more productive farms remaining in operation, a structural trend documented across the southeastern United States [12]. Such shifts may influence milk-quality trajectories by altering management practices, genetic composition, and production intensity among the remaining herds.

### Ethics statement

The dataset was de-identified and obtained under a data-sharing agreement with DHIA. All animal- and farm-level identifiers were removed before release, and no direct contact with animals or human subjects occurred. Consequently, this study was exempt from Institutional Review Board (IRB) and Institutional Animal Care and Use Committee (IACUC) review.

### Outcome variables

Three herd-level milk-quality indicators were analyzed as continuous outcomes:

1. **Somatic Cell Count (SCC):** Measured in thousands of cells per milliliter (×10^3^ cells/mL). Because the SCC distribution was right-skewed, log₁₀ transformation was applied before modeling. Back-transformed geometric means are reported for interpretability.
2. **Milk Fat (%):** The monthly average milk-fat percentage.
3. **Milk Protein (%):** The monthly average milk-protein percentage.

### Predictor variables

The following predictors were included to evaluate associations with milk-quality outcomes:

• **Year:** Modeled as a continuous variable to estimate linear temporal trends from 2011 to 2023.
• **VFD Policy Period:** Categorized as *Pre-VFD (2011–2015)*, *Transition (2016–2017)*, and *Post-VFD (2018–2023)*. Extended pre- and post-VFD periods captured baseline variability and delayed behavioral responses following implementation of the VFD. The transition interval (2016–2017) was treated separately to account for short-term management adjustments during the directive’s rollout.
• **Season:** Defined by meteorological convention: *Winter* (Dec–Feb), *Spring* (Mar–May), *Summer* (Jun–Aug), and *Fall* (Sep–Nov).

The study’s analytical design was guided by a conceptual framework (S1 File) linking policy, management, and environmental influences to milk-quality outcomes. The framework illustrates how the 2017 VFD may indirectly affect herd-level milk quality through management behaviors. These practices influence the cows’ micro-environment—such as stall cleanliness, ventilation, and heat-stress control—which, together with broader climatic conditions, determines milk composition and udder health. Solid arrows in the framework represent hypothesized associations, whereas dashed arrows denote indirect or untested pathways that provide contextual interpretation for observed trends.

### Statistical analysis

All analyses were conducted in R version 4.4.1 [13] using the lme4 [14], lmerTest [15], emmeans [16], ggplot2 [17], and tidyverse [18] packages.

Descriptive statistics summarized the distribution, central tendency, and variability of SCC, fat, and protein across years, policy periods, and seasons. Means, medians, and interquartile ranges were reported as appropriate.

SCC values were log₁₀-transformed to satisfy assumptions of normality and homoscedasticity. Statistical inferences were conducted on the transformed scale, with results back-transformed to geometric means for presentation. Corresponding 95% confidence intervals were computed from model estimates on the log scale. Milk fat and protein percentages met normality assumptions and were analyzed untransformed.

Trends were defined as systematic changes in mean values of each outcome (log₁₀SCC, fat %, protein %) over time, quantified by the fixed-effect slope for *Year* in linear mixed-effects models (LMMs). Seasonal variation was quantified using fixed *Season* effects with Tukey-adjusted pairwise contrasts to identify significant differences among categories.

Separate LMMs were fitted for each outcome variable. The LMM approach accounts for the hierarchical and unbalanced structure of the DHIA dataset, with repeated herd-level observations collected across years and variable participation durations.

Due to anonymization, individual farm identifiers were unavailable. To capture within-group correlation, herd-size category (based on the number of lactating cows) was modeled as a random intercept, representing production-level clustering.

Each model followed the general form


$$Y_{ijk}\;=\;\beta_{\text{0}}\;+\;\beta_{\text{1}}(Year)+\;\beta_{\text{2}}(Period)\;+\;\beta_{\text{3}}(Season)\;+u_{k}\;+\;\varepsilon_{ijk}$$

Where:

- *Y*_*ijk*_: Monthly outcome (logSCC, fat %, protein %) for herd-size group k
- *β*_*0*_: Overall intercept
- *β*_*1*_*, β*_*2*_*, β*_*3*_: Fixed effects for time, VFD period, and season
- *u*_*k*_: Random intercept for Size (herd size category), and
- ε_*ijk*_: Residual error term

Models were fitted using the function lmer(outcome ~ predictor + (1 | Size), data = df). Pairwise contrasts were estimated using emmeans with Tukey-adjusted *p*-values. Estimated marginal means and standard errors were reported.

The mixed-effects modeling approach follows established precedents for longitudinal dairy data [19,20], ensuring appropriate handling of repeated measures and unbalanced participation.

The conceptual framework (S1 File) informed analytical organization and interpretation but was not tested statistically; it provides context for understanding how policy, management, and environmental factors align with descriptive trends.

### Sample representativeness and limitations

The final dataset included 8,3study 57 monthly herd-level records. The median herd size was 249 lactating cows (mean = 320), indicating that most farms were medium-sized.

A limitation of this dataset is the absence of annual totals for all DHIA-enrolled farms, preventing formal assessment of representativeness. Consequently, we cannot quantify the proportion of Tennessee dairy farms that participate in DHIA, nor compare herd size or management practices between enrolled and non-enrolled operations. National data (e.g., USDA-NAHMS 2014) suggest that large operations may rely on internal testing, potentially leading to underrepresentation of the largest herds.

## Results

The analysis of 8,357 monthly herd-level records from Tennessee dairy farms (2011–2023) revealed significant and consistent improvements across all milk-quality parameters (SCC, milk-fat content, and milk-protein content. These trends were evident in long-term temporal analyses, across VFD policy periods, and within seasonal cycles. The long-term decline in SCC and concurrent rise in milk fat and protein is consistent with broader national reports of improved udder health and milk composition across U.S. dairy herds during the same period [1–3]. These sustained improvements reflect continued progress in herd management, milking hygiene, and selective breeding.

### Overview of temporal trends in milk quality (2011–2023)

**Fig 1:**
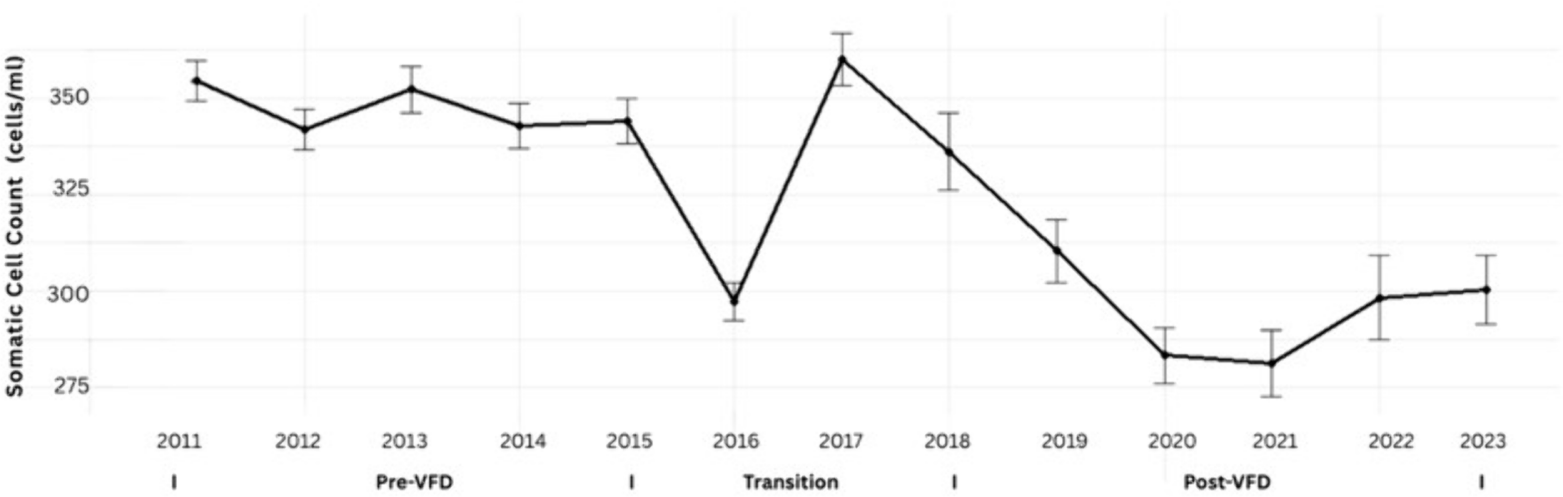
Annual trend in somatic cell count (SCC) among Tennessee DHIA herds, 2011–2023. Each point represents the mean annual SCC (×10^3^ cells/mL) aggregated from monthly herd-level observations across all participating Tennessee Dairy Herd Improvement Association (DHIA) herds. Vertical error bars denote standard errors of the mean. SCC values declined steadily after 2017, following a brief increase during the 2016–2017 transition period, indicating overall improvements in udder health among participating herds during the study period.

Across the 13-year study period, herd-level SCC declined steadily, indicating sustained improvements in udder health. After log₁₀-transformation to correct for skewness, the linear mixed-effects model estimated a negative slope of −0.0087 log₁₀ units per year (SE = 0.00070, 95% CI: −0.0101 to −0.0073; t = −12.44; p < 0.001), equivalent to an average annual reduction of roughly 2% in geometric mean SCC (Table 2). The fixed effect of Year remained highly significant in all model specifications, confirming the robustness of the trend. As illustrated in Fig 1, SCC declined consistently after 2017, following a brief plateau during the 2016–2017 transition period.

**Fig 2:**
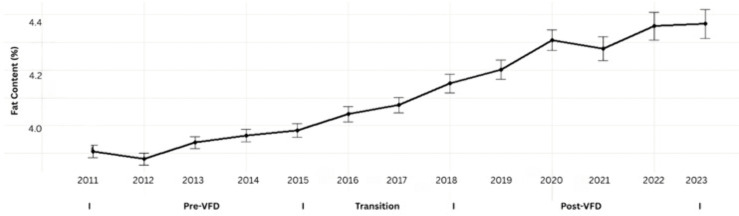
Annual trend in milk fat content among Tennessee DHIA herds, 2011–2023. Each point represents the mean annual milk-fat percentage aggregated from monthly herd-level data across all participating Tennessee Dairy Herd Improvement Association (DHIA) herds. Vertical error bars denote standard errors of the mean. Milk-fat content increased steadily throughout the study period, with sharper gains beginning after 2017. This sustained upward trajectory reflects long-term improvements in herd nutrition, genetic selection for milk components, and management efficiency rather than direct regulatory effects.

Milk-fat content exhibited a statistically significant upward trend throughout the study period (Fig 2). The model estimated an annual increase of 0.042 percentage points (95% CI: 0.0379–0.0469; t = 18.47; p < 0.001), corresponding to an overall rise from approximately 3.82% in 2011 to 4.25% in 2023.

**Fig 3:**
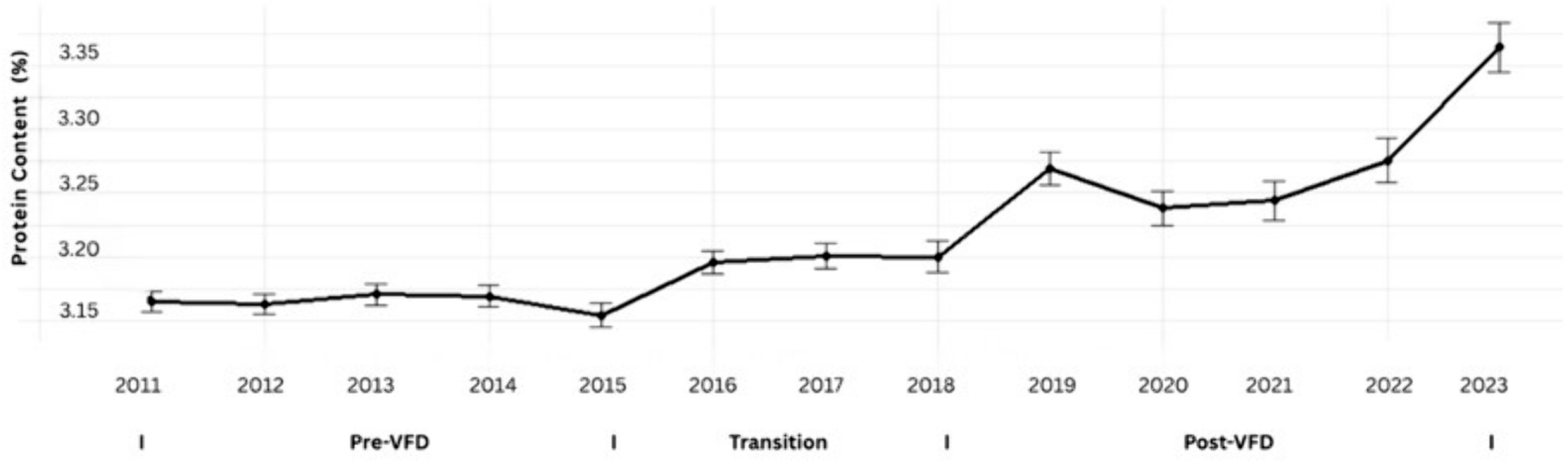
Annual trend in milk protein content among Tennessee DHIA herds, 2011–2023. Each point represents the mean annual milk-protein percentage aggregated from monthly herd-level data across all participating Tennessee Dairy Herd Improvement Association (DHIA) herds. Vertical error bars denote standard errors of the mean. Milk-protein content showed a gradual increase from 2011 through 2023, with slight acceleration during the post-2017 period. The steady upward trajectory reflects cumulative improvements in herd nutrition, genetics, and feed efficiency, consistent with national patterns of enhanced milk-component yields.

Milk-protein content also increased significantly during the same period (β = 0.0118; t = 13.80; p < 0.001). As shown in Fig 3, protein remained relatively stable in earlier years but rose consistently after 2017, paralleling national improvements in milk-component yields.

### Effects of the veterinary feed directive (VFD) on milk-quality parameters

To evaluate changes surrounding the 2017 implementation of the VFD (GFI #213), models were fitted with Period as a fixed effect: Pre-VFD (2011–2015), Transition (2016–2017), and Post-VFD (2018–2023) and herd-size category as a random intercept.

For SCC, the geometric mean declined from 311,170 cells mL ⁻ ¹ in the Pre-VFD period (log-mean = 2.493; SE = 0.0032) to 291,070 cells mL ⁻ ¹ during Transition (log-mean = 2.464; SE = 0.0058), and further to 264,850 cells mL ⁻ ¹ in the Post-VFD period (log-mean = 2.423; SE = 0.0046) (Table 1).

**Table 1:** Estimated marginal means for somatic cell count (SCC) across veterinary feed directive (VFD) implementation periods (2011–2023).

Period	logMean	SE	95% CI	Geometric Mean	SE	95% CI
Pre-VFD	2.493	0.0032	2.487–2.499	311.2	2.3	307.0–315.5
Transition	2.464	0.0058	2.453–2.475	291.1	4.2	283.9–298.5
Post-VFD	2.423	0.0046	2.414–2.432	264.9	3.3.	259.4–270.5

Geometric means calculated as 10^log₁₀Mean.

Pairwise contrasts confirmed significant reductions across all periods (Table 2), with the largest drop, 0.0698 log₁₀ units, or ≈ 17%, from Pre-VFD to Post-VFD (p < 0.0001).

**Table 2:** Pairwise comparisons of somatic cell count (SCC) between VFD implementation periods (2011–2023).

Contrast	Estimate (log₁₀)	SE	z-ratio	p-value	% Change
Pre-VFD – Transition	0.0292	0.0066	4.433	<0.0001	+6.9%
Pre-VFD – Post-VFD	0.0698	0.0056	12.510	<0.0001	+17.5%
Transition – Post-VFD	0.0406	0.0074	5.502	<0.0001	+9.9%

% Difference calculated from geometric means. All contrasts use Tukey adjustment.

Milk-fat content increased significantly over the same intervals (Tables 3, 4; Fig 2). The mean fat content rose from 3.89% (Pre-VFD) to 4.01% (Transition) and 4.22% (Post-VFD), with all contrasts statistically significant (p < 0.0001). The total increase of 0.33 percentage points represents an ≈ 8.5% relative gain in fat yield.

**Table 3:** Estimated marginal means for milk fat content across VFD policy periods.

Period	Estimated mean	SE	95% CI
Pre-VFD	3.89	0.0596	3.77–4.00
Transition	4.01	0.0616	3.89–4.13
Post-VFD	4.22	0.0604	4.10–4.33

**Table 4:** Pairwise comparisons of milk fat content between VFD policy periods.

Contrast	Estimate	SE	z-ratio	p-value	% Change
Pre-VFD vs. Transition	−0.124	0.0217	−5.702	<0.0001	+3.08%
Pre-VFD vs. Post-VFD	−0.329	0.0184	−17.911	<0.0001	+8.48%
Transition vs. Post-VFD	−0.206	0.0243	−8.475	<0.0001	+5.24%

Negative estimates indicate that the second period in the comparison had a higher mean fat content than the first. % Change is calculated as [(Mean Period2 – Mean Period1)/ Mean Period1] * 100. All contrasts use Tukey adjustment.

Similarly, milk-protein content increased from 3.14% (Pre-VFD) to 3.17% (Transition) and 3.23% (Post-VFD), with all pairwise contrasts significant (Tables 5, 6; Fig 3). The overall increase of 0.09 percentage points (≈ 2.9%) was modest but consistent with long-term management and nutritional progress rather than direct regulatory influence.

**Table 5:** Estimated marginal means for milk protein content across VFD policy periods.

Period	Estimated mean	SE	95% CI
Pre-VFD	3.14	0.0357	3.07–3.21
Transition	3.17	0.0362	3.10–3.24
Post-VFD	3.23	0.0359	3.16–3.30

**Table 6:** Pairwise comparisons of milk protein content between VFD policy periods.

Contrast	Estimate	SE	z-ratio	p-value	% Change
Pre-VFD vs. Transition	−0.0332	0.0081	−4.117	0.0001	+1.05%
Pre-VFD vs. Post-VFD	−0.0927	0.0068	−13.572	<0.0001	+2.95%
Transition vs. Post-VFD	−0.0596	0.0090	−6.603	<0.0001	+1.88%

Negative estimates indicate that the second period in the comparison had a higher mean protein content than the first. % Change is calculated as [(Mean Period2 – Mean Period1)/ Mean Period1] * 100. All contrasts use Tukey adjustment.

### Seasonal trends in milk quality parameters

Linear mixed-effects models demonstrated significant seasonal differences in all three outcomes (p < 0.001). Seasons were defined as Winter (Dec–Feb), Spring (Mar–May), Summer (Jun–Aug), and Fall (Sep–Nov).).

### Somatic cell count (SCC)

**Fig 4:**
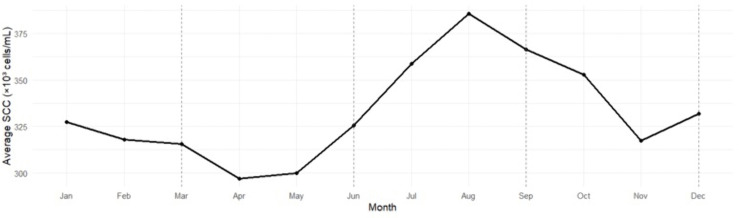
Seasonal trend in somatic cell count (SCC) among tennessee DHIA herds, averaged across 2011–2023. Each point represents the mean monthly SCC (×10^3^ cells/mL) aggregated from herd-level observations across all participating Tennessee Dairy Herd Improvement Association (DHIA) herds. Somatic cell counts were lowest during spring and early summer, increasing sharply through late summer and peaking in August, before declining again toward winter. This pattern reflects seasonal stressors such as elevated temperature–humidity, reduced cow comfort, and increased environmental bacterial exposure, which heighten mastitis risk and influence milk quality.

SCC peaked in Summer (314 × 10^3^ cells mL ⁻ ¹) and Fall (308 × 10^3^ cells mL ⁻ ¹) and was lowest in Spring (272 × 10^3^ cells mL ⁻ ¹) (Tables 7, 8; Fig 4). Pairwise contrasts confirmed that Spring SCC was significantly lower than both Summer (−0.0634 log₁₀ units; p < 0.001) and Fall (−0.0549 log₁₀ units; p < 0.001), equivalent to 13% and 12% reductions in geometric mean SCC.

**Table 7:** Seasonal patterns in SCC (2011–2023).

Season	log₁₀ Mean	SE	95% CI (log₁₀)	Geometric Mean
Winter	2.461	0.0047	2.452–2.470	289
Spring	2.434	0.0047	2.425–2.443	272
Summer	2.497	0.0049	2.488–2.507	314
Fall	2.489	0.0048	2.479–2.498	308

**Table 8:** Pairwise seasonal SCC comparisons.

Comparison	Δ (log₁₀ units)	SE	% Change	z-ratio	p-value
Winter vs. Spring	+0.0268	0.0066	+6.3%	4.034	0.0003
Winter vs. Summer	−0.0367	0.0068	−8.1%	−5.395	<0.0001
Winter vs. Fall	−0.0281	0.0067	−6.2%	−4.175	0.0002
Spring vs. Summer	−0.0634	0.0068	−13.0%	−9.353	<0.0001
Spring vs. Fall	−0.0549	0.0067	−11.9%	−8.162	<0.0001
Summer vs. Fall	+0.0085	0.0069	+2.0%	1.236	0.6040

All contrasts Tukey-adjusted. % Change = (10^Δ – 1)100. Positive Δ indicates higher SCC in first season.

### Milk fat content

**Fig 5:**
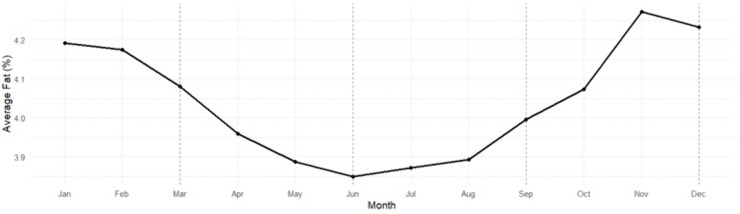
Seasonal trend in milk fat content among Tennessee DHIA herds, averaged across 2011–2023. Each point represents the mean monthly milk-fat percentage aggregated from herd-level observations across all participating Tennessee Dairy Herd Improvement Association (DHIA) herds. Milk fat content reached its lowest values during summer months (June–July) and peaked during late autumn and winter. The cyclical pattern reflects the combined influence of temperature stress, feed intake, and milk-yield dynamics—where heat stress and reduced dry-matter intake during summer decrease fat synthesis, while cooler conditions and improved feed efficiency during winter promote higher fat concentration.

Seasonal variation in milk-fat percentage was also significant (Tables 9, 10; Fig 5). Fat content reached its lowest values during Summer (3.84%) and peaked in Winter (4.17%), with a total seasonal amplitude of ≈ 0.33 percentage points. Winter milk contained 8.6% more fat than Summer (p < 0.0001), consistent with reduced feed intake and increased heat load during warmer months.

**Table 9:** Seasonal patterns in Milk Fat (2011–2023).

Season	Estimated mean (%)	SE	95% CI
Winter	4.17	0.0456	4.08-4.26
Spring	3.95	0.0455	3.86-4.03
Summer	3.84	0.0458	3.75-3.93
Fall	4.08	0.0457	3.99-4.17

**Table 10:** Pairwise seasonal milk fat comparisons.

Comparison	Difference	SE	% Change	z-ratio	p-value
Winter vs. Spring	+0.2219	0.0218	+5.6%	10.167	<0.0001
Winter vs. Summer	+0.3296	0.0224	+8.6%	14.739	<0.0001
Winter vs. Fall	+0.0912	0.0222	+2.2%	4.109	0.0002
Spring vs. Summer	+0.1077	0.0223	+2.8%	4.825	<0.0001
Spring vs. Fall	−0.1308	0.0221	−3.3%	−5.906	<0.0001
Summer vs. Fall	−0.2384	0.0227	−6.2%	−10.519	<0.0001

### Milk protein content

**Fig 6:**
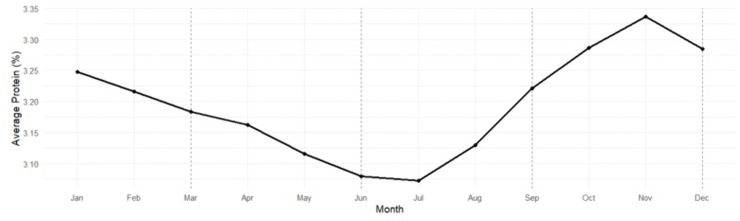
Seasonal trend in milk protein content among Tennessee DHIA herds, averaged across 2011–2023. Each point represents the mean monthly milk-protein percentage aggregated from herd-level data across all participating Tennessee Dairy Herd Improvement Association (DHIA) herds. Milk protein content reached its lowest levels during summer (June–July) and peaked in late autumn and early winter (October–December). This seasonal pattern reflects physiological and nutritional dynamics, where heat stress and reduced dry-matter intake during summer depress milk solids, while cooler temperatures, improved feed efficiency, and higher forage quality during winter support increased protein synthesis.

Protein content showed a similar seasonal cycle (Tables 11, 12; Fig 6), lowest in Summer (3.07%) and highest in Fall (3.25%) and Winter (3.22%). The Summer–Fall contrast (−0.187 percentage points; p < 0.0001) represented a ≈ 5.5% seasonal decline, mirroring the thermally driven reduction in milk solids.

**Table 11:** Seasonal patterns in milk protein (2011–2023).

Season	Estimated mean (%)	SE	95% CI
Winter	3.22	0.0317	3.16–3.29
Spring	3.13	0.0317	3.07–3.19
Summer	3.07	0.0317	3.01–3.13
Fall	3.25	0.0317	3.19–3.32

**Table 12:** Pairwise seasonal milk protein comparisons.

Comparison	Difference	SE	% Change	z-ratio	p-value
Winter vs. Spring	+0.096	0.0079	+2.88%	12.221	<0.0001
Winter vs. Summer	+0.157	0.0081	+4.89%	19.541	<0.0001
Winter vs. Fall	−0.030	0.0080	−0.92%	−3.738	0.0011
Spring vs. Summer	+0.061	0.0080	+1.95%	7.629	<0.0001
Spring vs. Fall	−0.126	0.0080	−3.69%	−15.796	<0.0001
Summer vs. Fall	−0.187	0.0082	−5.54%	−22.939	<0.0001

### Model performance and assumptions

All mixed-effects models satisfied assumptions of normality and homoscedasticity. The inclusion of herd-size category as a random intercept effectively accounted for within-group clustering, while fixed effects for Year, VFD period, and Season captured the major temporal and environmental sources of variation. These results confirm that observed improvements in milk quality reflect robust, long-term patterns rather than short-term fluctuations.

## Discussion

This study described temporal and seasonal patterns in milk-quality indicators: somatic cell count (SCC), milk fat, and milk protein among Tennessee dairy herds from 2011 to 2023, and interpreted observed changes in relation to the 2017 implementation of the VFD. Guided by the conceptual framework (S1 File), our analyses emphasize descriptive associations rather than causal inference. The framework hypothesizes that policy may act indirectly, through management behaviors such as veterinary engagement, milking hygiene, and antimicrobial stewardship—which, in turn, influence the environmental and physiological conditions shaping milk quality. Although direct measures of management and environment were not available, the temporal and seasonal signals provide population-level indicators of change, not evidence of a direct pharmacological effect of the VFD on milk composition.

### Trends in milk quality parameters

We observed statistically significant improvements in milk quality across Tennessee between 2011 and 2023: SCC declined while milk fat and protein increased. These findings parallel national and international reports of sustained progress in udder health and milk composition [21–24] including reductions in SCC and clinical mastitis attributed to multi-year investments in mastitis-control programs, milking hygiene, and genetic selection [2,25–27]. Similar progress has been reported in the United States, where bulk-tank SCC declined substantially from 1999 to 2019 [3,28].

Within Tennessee, the most likely explanation interpretation is that these trends reflect cumulative management and structural advances rather than a discrete policy effect. Adoption of precision herd-management tools, nutrition programs targeting components, and selective breeding for production and health traits likely underlie the observed improvements [22,26,27]. Payment systems that reward low SCC and higher component yields further incentivize practices that elevate milk quality [29]. Particularly, while the legal SCC limit under the Pasteurized Milk Ordinance remained at 750,000 cells/mL throughout the study period, most processors imposed stricter thresholds: often ≤400,000 cells/mL and withheld premiums or imposed penalties for non-compliance. Similarly, component-based pricing for fat and protein became more prevalent after 2015, independently incentivizing higher solids. These market forces, unrelated to the 2017 VFD, likely contributed to the observed improvements in milk quality. Together, these drivers support the conclusion that progressive improvements in management, genetics, and market alignment, rather than regulatory change alone, best explain the steady enhancement of milk quality during the study period.

### Influence of the veterinary feed directive (VFD) implementation

Temporal coincidence between the 2017 VFD and continued improvements in milk quality should be interpreted cautiously. Although the post-VFD period (2018–2023) coincided with lower SCC and modest gains in components, the directive targeted medically important antimicrobials administered in feed or water, primarily affecting non-lactating cattle and did not alter feed formulations or intramammary antimicrobial use in lactating cows [8]. Field studies in Tennessee and Ohio indicate that the VFD’s most tangible effects were administrative (e.g., discontinuation of medicated milk replacers for calves; enhanced veterinary oversight), not biological modifications to lactating-cow diets or therapies [30,31].

Moreover, most antimicrobial use relevant to udder health (clinical and dry-cow therapy) was not directly changed by the VFD [32–34]. national surveillance shows stable sales of intramammary products over the past decade [9,34]. Taken together, these observations support the view that post-2017 gains likely reflect ongoing management, nutritional, and genetic progress, with any VFD-related influence operating indirectly, by reinforcing veterinary oversight, and preventive herd-health practices rather than through direct pharmacological restriction (S1 File).

### Seasonal variations in milk quality

Seasonal patterns were biologically consistent across outcomes. SCC peaked in summer and declined in winter, consistent with heat stress, elevated environmental bacterial load, and reduced cow comfort [35,36]. High temperature, humidity impairs immune function and increases mastitis risk, while lower summer milk yield can concentrate SCC in bulk milk [37–40]. In contrast, milk fat and protein were highest in winter and lowest in summer patterns reflecting thermal load, feed-intake dynamics, and milk-yield dilution effects [26,27].

Although milk yield was not directly evaluated in this study, established evidence indicates that changes in milk output influence milk-component concentrations through both dilution and metabolic mechanisms. Periods of higher milk yield, typically occurring during warmer months or under improved nutritional conditions, are often associated with lower fat percentage due to increased secretion volume and altered nutrient partitioning in the mammary gland [41,42]. Conversely, cooler months tend to coincide with slightly lower yield but higher concentrations of fat and protein, reflecting both physiological concentration effects and improved energy balance. These established yield–composition dynamics provide a biological explanation for the seasonal variation observed in Tennessee herds, reinforcing those environmental and physiological drivers, rather than direct regulatory factors, explain much of the component variability.

Cooler conditions promote feed intake and metabolic efficiency, supporting higher solids. Winter rations often include preserved forages that enhance rumen fermentation and fat synthesis [6]. Moreover, shorter photoperiods may favor hormonal profiles conducive to component yield [36,43]. These seasonal signals underscore practical opportunities to stabilize quality (e.g., cooling and ventilation, ration adjustments to maintain energy balance, and timing of peak lactation).

### Integrated interpretation and management implications

Synthesizing these results, improvements in Tennessee milk quality from 2011 to 2023 are most consistent with sustained progress in herd management, udder-health programs, nutrition, and genetic selection, rather than direct effects of the VFD. Industry dynamics likely reinforced these trajectories. The USDA APHIS documented national declines in bulk-tank SCC between 1999 and 2019 [3]. Similar improvements have been linked to market and structural drivers operating independently of regulatory policy. Many processors or cooperatives imposed buyer thresholds (often ≤400,000 cells/mL) and component-based pricing that reward higher fat and protein [5,21–26]. Such market incentives, operating alongside producer-driven management changes, plausibly contributed to the observed trends independent of federal antibiotic policy.

Seasonal variation emphasizes the continuing importance of environmental management. Cooling, hygiene, and ration strategies can reduce the drop in milk quality during summer. From a systems perspective, the descriptive trends observed here illustrate how policy interacts with broader structural and behavioral forces. As depicted in the conceptual framework (S1 File), regulatory measures are most effective when aligned with producer-led improvements in management, technology adoption, and animal welfare. Continued investment in producer education, precision-dairy tools, and antimicrobial stewardship will be essential to sustain progress.

Finally, structural consolidation likely influenced aggregate outcomes. In the southeastern United States, dairy farm numbers declined by ~39% between 2008 and 2017, while average herd size and per-cow productivity increased [12]. Selection for more efficient, better-managed herds could contribute to declining SCC and improving solids in the analytic cohort, independent of policy effects, and should be considered when interpreting temporal trends.

Sustaining gains in milk quality will require both biological resilience and system-level adaptation, aligning policy, management, and market incentives under changing climatic and economic conditions.

## Conclusion

This study provided a longitudinal assessment of milk-quality trends in Tennessee dairy farms from 2011 to 2023. It revealed steady improvements in SCC, fat, and protein content alongside predictable seasonal patterns. These results reflect sustained advancements in herd management, animal health, and environmental adaptation rather than direct regulatory effects of the VFD. While policy frameworks such as the VFD may reinforce veterinary oversight and stewardship, the dominant influences on milk quality remain management efficiency, environmental control, and genetic progress. Continued coordination among producers, veterinarians, and policymakers will be essential to maintain these gains and ensure the resilience of dairy production under evolving climatic and regulatory conditions.

## Supporting information

S1 File Conceptual framework illustrating hypothesized relationships among policy, management, environment, and milk-quality outcomes. The diagram shows the proposed relationships between the Veterinary Feed Directive (policy), management practices (genetics, nutrition, hygiene, and disease prevention), environmental factors (season, housing and ventilation, and extreme weather conditions), and milk-quality outcomes (somatic cell count, milk fat, and milk protein). Solid arrows represent pathways supported by literature, while dashed arrows indicate hypothesized pathways not measured in this dataset.
(TIF)
